# Genetic admixture predicts parasite intensity: evidence for increased hybrid performance in Darwin's tree finches

**DOI:** 10.1098/rsos.181616

**Published:** 2019-04-03

**Authors:** Katharina J. Peters, Christine Evans, J. David Aguirre, Sonia Kleindorfer

**Affiliations:** 1 College of Science and Engineering, Flinders University, Adelaide, Australia; 2 School of Natural and Computational Sciences, Massey University, Auckland, New Zealand; 3 Konrad Lorenz Research Station and Department of Behavioural Biology, University of Vienna, Austria

**Keywords:** hybrid fitness, adaptive capacity, *Philornis downsi*, Galápagos, parasites, *Camarhynchus*

## Abstract

Hybridization can increase adaptive potential when enhanced genetic diversity or novel genetic combinations confer a fitness advantage, such as in the evolution of anti-parasitic mechanisms. Island systems are especially susceptible to invasive parasites due to the lack of defence mechanisms that usually coevolve in long-standing host–parasite relationships. We test if host genetic admixture affects parasite numbers in a novel host–parasite association on the Galápagos Islands. Specifically, we compare the number of *Philornis downsi* in nests with offspring sired by Darwin's small tree finch (*Camarhynchus parvulus*), Darwin's medium tree finch (*C. pauper*) and hybrids of these two species. The number of *P. downsi* decreased with an increasing genetic admixture of the attending male, and nests of hybrid males had approximately 50% fewer parasites than *C. parvulus* nests, and approximately 60% fewer parasites than *C. pauper* nests. This finding indicates that hybridization in this system could be favoured by selection and reveal a mechanism to combat an invasive parasite.

## 1. Introduction

Introduced parasites can wreak havoc on native hosts, especially in island systems where naive hosts lack defensive anti-parasite mechanisms [1]. The Red Queen Hypothesis proposes that hosts are selected to constantly adapt to rapidly evolving parasites, and hence novel genetic recombinations that facilitate adaptation in the host can present a fitness advantage under conditions of parasitism. One mechanism to increase genetic diversity in a host is genetic introgression via hybridization [2]. Adaptation to an introduced parasite through hybrid vigour presents a hypothesis for the occurrence of hybridization, hence recognizing hybrid vigour in systems with introduced parasites is a crucial step towards understanding the extensive occurrence of hybridization worldwide and its evolutionary role in host–parasite systems [3].

The Galápagos Islands (hereafter Galápagos) are currently being invaded by a suite of species that span several taxonomic groups and pose extinction threats to the endemic flora and fauna (e.g. cottony cushion scale *Icerya purchasi*, black rat *Rattus rattus* and smooth-billed ani *Crotophaga ani*) [4–6]. Land birds are particularly threatened by the introduced fly *Philornis downsi* (Diptera: Muscidae) [4], whose females oviposit in avian nests where their larvae consume the blood and tissue of nestlings, causing naris malformation [7], blood loss, as well as external and internal wounds [8] with up to 100% annual brood loss through direct consumption [9]. After feeding on the nestlings for approximately 10 days, larvae pupate in the base of the nest and emerge as imagines after approximately 15 days [9]. *Philornis downsi* is considered the biggest risk factor for the extinction of the two critically endangered Darwin's finch species (Passeriformes: Thraupidae), the mangrove finch (*Camarhynchus heliobates*) on Isabela Island [10] and the medium tree finch (*C. pauper*) on Floreana Island [11,12].

*Camarhynchus pauper* hybridizes with the small tree finch (*C. parvulus*) [13], which is driven by matings between female *C. pauper* and male *C. parvulus* [14]. Genetic analyses showed that in some years, 60% of captured adult tree finches were hybrids, likely extending past the F1 generation [14]. In light of the introduced parasite, Kleindorfer *et al*. [13] and Peters *et al*. [14] raised the possibility of increased hybrid fitness in this system. Previous studies have found fewer *P. downsi* in nests of *C. parvulus* than in those of *C. pauper* nests, with differing nest heights among species (*C. pauper* highest, *C. parvulus* lowest) suspected as a factor [9]. Furthermore, it was shown that higher nests had more *P. downsi* [15].

Here, we examine drivers of variation in *P. downsi* numbers among *Camarhynchus* nests by examining the relationship between the genetic admixture of the sire for each nest and the number of parasites in each nest. We examine the number of *P. downsi* per nest in relation to the genetic admixture of the attending male because males build the nest in this system and males can be sampled and colour-banded at the onset of the nesting phase, while nestlings are unfeasible to access due to the high nest locations in this species. Specifically, we use molecular approaches in combination with field observation to quantify differences in the magnitude of *P. downsi* infestation in nests built by males of the two parental tree finch species and hybrid males on Floreana Island.

## 2. Material and methods

### 2.1. Study species and study site

We collected data in the *Scalesia* forest at the base of the Cerro Pajas volcano (1°17 S, 90°27 W, elevation 300–400 m, [11]) on Floreana Island, Galápagos, from January to April in 2010, 2013 and 2014 using our long-term field monitoring protocol [16]. *Camarhynchus* males build a display nest to attract a female [17]. Females produce clutches of 2–4 eggs that they incubate for approximately 14 days. At the onset of the nesting phase, we used mist-nets to capture, mark and genetically sample nest builders (described below). We monitored nesting activity of banded adult males in three Darwin's tree finch groups: small tree finch (*C. parvulus*, approx. 12 g), medium tree finch (*C. pauper,* approx. 16 g) and their hybrid (approx. 13 g) [13,14]. We monitored nesting activity by checking each nest every three days. From the 10th day of incubation onwards, we checked nests every two days so we would not miss the hatching event which usually occurs around day 14. Using a ladder and a pole mounted scope camera, we were able to accurately record the nest contents. After the nesting events had finished, we collected and dismantled the nests and counted *P. downsi* larvae, pupae and empty puparia and assessed larval stages (first, second and third instar) using criteria outlined in [18] and [15].

### 2.2. Genetic admixture

Darwin's tree finches nest from heights of 2 m, but nests are rarely below 4 m and often at heights of up to 10 m in the slender and delicate *Scalesia pedunculata*, which makes it unfeasible to extract the nestlings for genetic sampling. Additionally, catching the females is extremely difficult as they rarely descend low enough for mist netting and, unlike males, do not respond to acoustic stimuli once paired. We therefore analysed *P. downsi* numbers in relation to the male's genetic composition because (1) males build the nest to attract females for nesting [17], (2) *Camarhynchus* females inspect a nest and the male and choose a nest and mate [17], (3) occasionally *C. pauper* females choose a heterospecific male (usually a hybrid) but most *Camarhynchus* finches (including hybrids) pair with a mate of their own genetic group [14], and (4) therefore the male of the nest is a good predictor of the female's species ID.

We used nine microsatellite loci to assign adult males to three genetic groups (*C. parvulus*, *C. pauper*, hybrid) based on the individual membership coefficient (*q*_i_) derived from Bayesian clustering analysis using STRUCTURE [19], which rates the probability (0–1) of an individual belonging to the *C. parvulus* cluster (*q*_i_ ≥ 0.80 for *C. parvulus*, *q*_i_ ≤ 0.20 for *C. pauper* and 0.80 >*q*_i_ > 0.20 for the hybrid group) [14]. All loci were unlinked and confirmed to be neutral with a mean of 9.2 ± 1.3 alleles per locus and 0.54 ± 0.07 mean expected heterozygosity. We conformed the *q*_i_ threshold via analyses of simulated data using HYBRIDLAB ([20], but see [14] for detailed methods.)

To analyse the relationship between parasite numbers across tree finch nests and genetic admixture, we used *q*_i_ to calculate a hybrid index (HI): we retained the *q*_i_ for individuals with *q_i_* < 0.50 and used the inverse value (1−*q_i_*) for individuals with *q_i_* > 0.50. The highest HI value was 0.5 (0.5 probability to belong to either of the two clusters = highest degree of admixture possible) and the lowest value 0 (1.0 probability to belong to one of the two clusters = no genetic admixture).

### 2.3. Statistical analysis

To avoid pseudoreplication, we only included the first nesting event observed for each male and excluded subsequent nests within and across years. We further excluded nests that failed at the egg stage, since these generally do not contain *P. downsi* larvae (but see [21]), resulting in a total of 27 nests (*C. parvulus* = 4, *C. pauper* = 5, hybrid = 18).

We constructed a generalized linear mixed model (implemented using the R package lme4 [22], assuming a Poisson error distribution and log link), to quantify the drivers of variation in *P. downsi* numbers among Darwin's finch nests. To account for potentially confounding relationships introduced by the different brood sizes, nest heights and number of days the nestlings survived, we estimated these effects in the same model we used to estimate the relationship between parasite numbers and the hybrid index. For nest height and the number of nestlings, we assumed a deterministic effect on parasite numbers [15,23]. For the days the nestlings survived, we were unsure of the directionality of the effect. Accordingly, we used a categorical dummy variable, which we called ‘nest trait’, that indexed the data for parasite number and the number of days the nestlings survived. The response vector for our analyses contained the counts for parasite number and the number of days nestlings survived. The fixed categorical predictors were nest trait (NT, two levels) and year (Yr, three levels). The fixed continuous predictors were the hybrid index (HI), nest height (NH) and brood size (BS), as well as the interactions between the continuous predictors and nest trait. Lastly, to account for the correlation between parasite number and the number of days the nestlings survived, the nest was included as a random effect allowing random intercepts for the two levels of the nest trait variable and a symmetric covariance structure.

To test the significance of the fixed effects we used backwards model selection based on a series of nested log-likelihood ratio tests (table 1). We started by examining the significance of the interaction terms, comparing a reduced model without a particular interaction term to the full model (model 1; table 1). Once the best-supported model for the interactions was identified (model 5; table 1) we then examined the significance of the main effects terms, excluding those involved in significant interactions, by comparing the reduced model of the main effects to the best-supported model for the interactions. This procedure allowed us to determine the model with the fewest number of parameters that best predicted the probability of our data (model 9, table 1). For all models, the random effects specification remained the same as described above. To test the robustness and convergence of our parameter estimates, we confirmed that they were consistent across available optimization routines for each model. Last, we confirmed that overdispersion was not apparent in our best-supported model using a *χ*^2^-test based on the ratio of the sum of squared Pearson residuals and the residual degrees of freedom ($\chi_{47}^{2}=19.28,$ *p* = 0.99).

**Table 1.** To determine the best-supported model for the number of parasites in Darwin's finch nests, we used a backward model selection based on nested log-likelihood ratio tests. In the candidate models, *Y* is the response vector containing the values for the number of parasites and the number of days the nestlings survived, NT is the nest trait variable considered at two levels, HI is the hybrid index, Yr is the year, BS is the brood size and NH is the nest height. Model 1 is the full model, and model 9 is the model best supported by our data.

model no.	model terms
1	*Y* = NT + HI + Yr + BS + NH + NT : HI + NT : NH + NT : BS
2	*Y* = NT + HI + Yr + BS + NH + NT : HI + NT : NH
3	*Y* = NT + HI + Yr + BS + NH + NT : HI + NT : BS
4	*Y* = NT + HI + Yr + BS + NH + NT : NH + NT : BS
5	*Y* = NT + HI + Yr + BS + NH + NT : HI
6	*Y* = NT + HI + Yr + BS + NT : HI
7	*Y* = NT + HI + Yr + NH + NT : HI
8	*Y* = NT + HI + BS + NH + NT : HI
9	*Y* = NT + HI + NT : HI

To further ensure our results reflected the effect of genetic admixture (measured as hybrid index) on parasite numbers, we used a simple generalized linear model to examine if the significant negative relationship between hybrid index and parasite number remained when we considered only the nests sired by hybrid males.

## 3. Results

**Figure 1.**
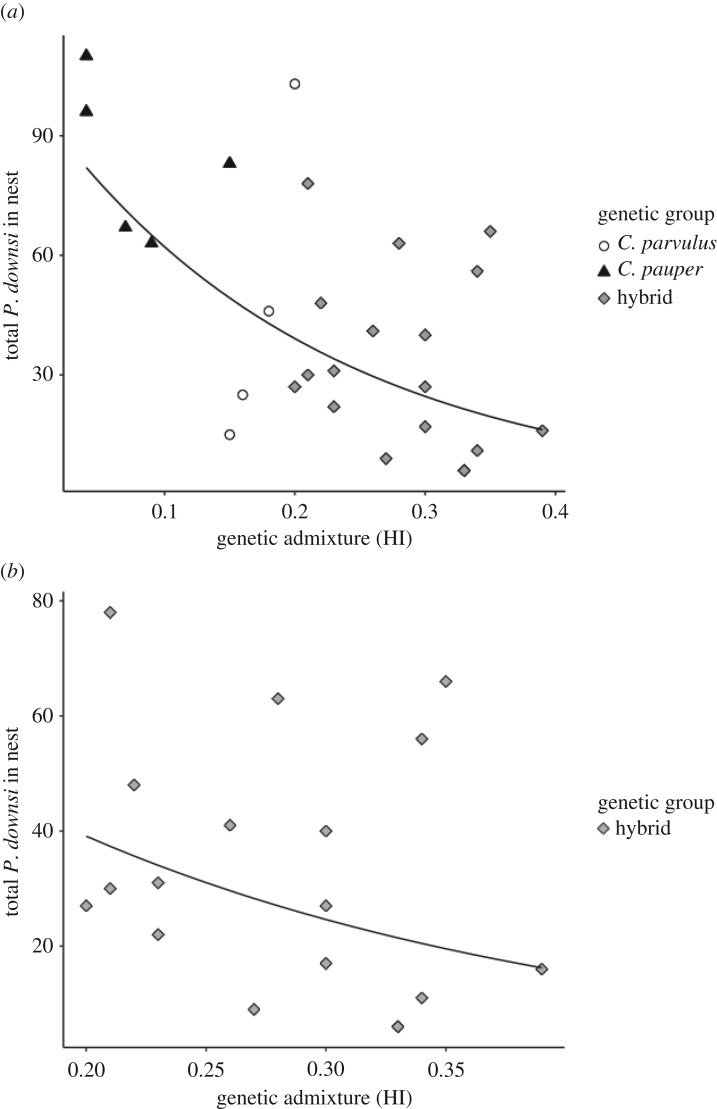
The relationship between the number of *P. downsi* larvae per nest and genetic admixture of associated male Darwin's tree finches (*Camarhynchus* spp.) on Floreana Island, Galápagos, with (*a*) showing both the relationship across nests sired by the parental species (*C. pauper* and *C. parvulus*) and hybrids, and (*b*) showing the relationship only for nests sired by hybrid males. Genetic admixture was measured using a hybrid index (HI), derived from a membership coefficient of microsatellite data. Although we denote the genetic group of the male that sired the nest in the figure, these are for illustrative purposes only as all our analyses and inferences are based on the HI with a sample size of 27 males with nests.

Overall, *P. downsi* numbers per nest decreased with an increasing genetic admixture of the host male (measured using HI), regardless of the male's genetic group (table 2 and figure 1*a*). Nestlings sired by hybrid males suffered from fewer parasites compared to nestlings sired by *C. parvulus* and *C. pauper* males (table 2 and figure 1*a*). Furthermore, examining the subset of the data containing only hybrid males we found that the significant negative relationships between the hybrid index and the number of parasites remained (Wald *Z*_1,17_ = 20.475, *p* < 0.001, figure 1*b*). Moreover, year, the number of nestlings or nest height did not affect *P. downsi* numbers (table 2). Using the average hybrid index for each group as a guide, hybrid nests had approximately half the number of parasites (26) as *C. parvulus* nests (41 *P. downsi* per nest), and one-third of the number of parasites as *C. pauper* nests (59 *P. downsi* per nest) (figure 1).

**Table 2.** Summary tables for the generalized linear mixed model (GLMM) examining the relationship between parasite number and the hybrid index, conditional on the effects of year, number of nestlings, nest height and the days nestlings survived. (*a*) Nested log-likelihood ratio test results for the models described in table 1. (*b*) Treatment contrasts table where the number of parasites was set as the reference category for the variable ‘Nest trait’ allowing us to test the hypothesis that the slope of the relationship between hybrid index and the number of parasites was significantly different from zero. *χ*^2^ log-likelihood ratio tests (LRT) are an appropriate test for GLMMs without overdispersion [24], as was the case for the models considered here (Material and methods).

	−2LL	d.f.	LRT
(*a*)			
*full model*			
model 1	355.83	13	
*testing the significance of the interactions*			
model 2	355.84	12	$\chi_{1}^{2}=0.001,$ *p* = 0.965
model 3	356.38	12	$\chi_{1}^{2}=0.546,$ *p* = 0.460
model 4	359.99	12	$\chi_{1}^{2}=4.152,$ *p* = 0.042
reduced model for the interaction effects			
model 5	356.44	11	
*testing the significance of the main effects*			
model 6	356.73	10	$\chi_{1}^{2}=0.284,$ *p* = 0.594
model 7	356.45	10	$\chi_{1}^{2}=0.005,$ *p* = 0.945
model 8	361.36	9	$\chi_{1}^{2}=4.913,$ *p* = 0.086
*best-supported model*			
model 9	362.79	7	

contrast	estimate	Wald *Z*	*p*
(*b*)			
number of parasites	4.591	14.043	<0.001
number of days	−2.302	−7.567	<0.001
number of parasites: hybrid index	−4.627	−3.459	<0.001
number of days: hybrid index	2.893	2.278	0.023

## 4. Discussion

Novel parasites can exert strong selective pressure on naive hosts [25]. Here, we show that nests sired by hybrid tree finch males with the highest genetic admixture had the lowest number of *P. downsi* larvae and pupae, while nests sired by either parental species had more *P. downsi*. This finding is an important step towards understanding this newly evolving host–parasite system and the importance of genetic diversity in host–parasite associations.

Hybrid fitness in relation to parasites varies across taxa (reviewed in [26]). Host genetic diversity can enhance resistance against disease and parasites (reviewed in [27]), but mechanisms vary. Most studies of hybrid vigour have been carried out in laboratory settings (e.g. Hawaiian silverswords [28], sockeye salmon [29] and *Drosophila* [30]) and therefore explored genetic rather than ecological components of hybrid fitness [31]. For example, Moulia *et al.* [32] found fewer intestinal pinworm *Aspiculuris tetraptera* in hybrids of *Mus musculus domesticus* and *M. m. musculus*, which the authors interpret as a consequence of genetic recombination. Grant & Grant [33] determined that ecological variables influence hybrid fitness in their study of hybrids between Darwin's medium ground finches (*Geospiza fortis*) and Darwin's cactus finches (*G. scandens*); here, hybrid fitness depended on variability in the size of seeds available.

Genetic diversity is particularly limiting for small and endangered populations with a depleted gene pool [34], and hybridization has been long recognized as an ‘evolutionary stimulus' generating bursts of evolutionary activity [35]. While the cause of hybridization in the case of Darwin's tree finches may be a lack of available mates for *C. pauper* due to continuing population decline [12,14], selection could favour the novel genotypes for different reasons, such as enhanced survivorship due to behavioural or ecological differences.

In this study, nest height was not associated with parasite numbers, in contrast to findings from a study on the two parental species *C. parvulus* and *C. pauper* [15]. This raises questions about why the inclusion of hybrid birds would remove the effect on the number of *P. downsi* per nest. Our study did not confirm the previously observed effect of nest height on parasite intensity; instead, we showed that genetic admixture was a far stronger predictor of *P. downsi* numbers even after accounting for the differences in nest height among hybrids and the pure parental populations. In addition to novel genetic combinations from introgression, hybrid birds had intermediate foraging height [36] and nest height [37] and may also express novel anti-parasite behaviours. Birds have a range of anti-parasite behaviours [38] to remove ectoparasites (e.g. feather lice in birds [39] or ticks in ungulates [40]). Using video in-nest camera monitoring, O'Connor *et al*. [41] observed parent finches removing *P. downsi* larvae from their nestlings, as well as parasite-related preening behaviour by the nestlings themselves. We currently do not know whether the genetic groups differ in these behaviours, but if hybrid parents are more vigorous at preening larvae from their nestlings, or hybrid nestlings display increased preening behaviour, this could potentially lower parasite numbers in hybrid nests.

Little is known about the ecology, behaviour and host selection criteria of *P. downsi* on the Galápagos. Kleindorfer and Dudaniec previously found that larger nests and nests in close nesting aggregations had higher *P. downsi* numbers [42]. In the case of mobile parasites infecting multiple hosts, social nesting aggregations can increase the detectability of host nests and therefore result in higher infestation levels than solitary nests [43], as has been shown in this system on Santa Cruz Island [42]. The highland forest is the main habitat for *Camarhynchus* tree finches on Floreana Island and also harbours two *Geospiza* ground finches; finch nesting density is higher in the highlands than lowlands. On Floreana Island, Dudaniec *et al.* [44] found that nesting habitat was associated with *P. downsi* infestation behaviour and offspring genetic relatedness. Female *P. downsi* oviposited a greater percentage of their clutch in lowland hosts nests and those nests had higher *P. downsi* offspring genetic relatedness, while in highland habitat, female *P. downsi* oviposited fewer eggs per clutch but they infected a greater number of host nests [44]. Future research should examine nesting density in *Camarhynchus* tree finches with a specific focus on the relative spatial distribution of hybrid nests in *Scalesia* forest on Floreana Island. Perhaps highland hybrid nests are more solitary, for example, which could explain lower parasite intensity.

In addition to visual cues, parasitic insects may use olfactory cues for host selection (e.g. [45]). We currently do not know the importance of host odour for host nest selection by adult *P. downsi*. If odour does play a significant role, differences between *C. parvulus*, *C. pauper* and the hybrid birds may be another factor that explains differential oviposition by *P. downsi* females. Based on the findings of this study, perhaps hybrid tree finch odour cues are less attractive to *P. downsi* females, and for this reason, fewer flies oviposit in hybrid nests.

Given the strong signal of genetic admixture for a number of *P. downsi* larvae per nest, our findings suggest that the Darwin's finch and *P. downsi* system will be useful to discover molecular pathways for anti-parasite outcomes. Future research should aim to identify such pathways that confer immune defense against *P. downsi*. These may regulate immunogenetic response and/or generate novel behavioural responses associated with novel genetic architecture.

## Data accessibility

Data available from the Dryad Digital Repository: https://doi.org/10.5061/dryad.j1st8p8 [46].
